# Prediction of gas production rate from shale gas reservoirs using a micro–macro analysis

**DOI:** 10.1038/s41598-023-27745-7

**Published:** 2023-01-10

**Authors:** Dantong Lin, Di Zhang, Xinghao Zhang, Bruno M. Goncalves da Silva, Liming Hu, Jay N. Meegoda

**Affiliations:** 1 Department of Civil & Environmental Engineering, New Jersey Institute of Technology, Newark, USA; 2 State Key Laboratory of Hydro-Science and Engineering, Department of Hydraulic Engineering, Tsinghua University, Beijing, China

**Keywords:** Civil engineering, Energy infrastructure

## Abstract

Shale gas has become one of the important contributors to the global energy supply. The declining pattern of the gas production rate with time from an unconventional gas reservoir is due to the depletion of shale gas stored in the nanovoids of the shale formation. However, there are only limited ways to predict the variation of the gas production rate with time from an unconventional gas reservoir. This is due to the multiple transport mechanisms of gas in nano-scale pores and changes in shale gas permeability with pressures in nano-scale pores, which is impacted by the pore structure of the shale. In this study, the permeability-pressure (*K*-*p*) relationship for different shales (Eagle Ford, Haynesville, Longmaxi and Opalinus) were determined using an equivalent anisotropic pore network model (PNM). This PNM has REV-scale shale gas flow in randomly generated nanovoids and their connection in the shale matrix, and the multiphase flow of shale gas including viscous flow, slip flow and Knudsen diffusion. These predicted *K*-*p* correlations were then used in a finite element model (FEM) to predict the variation of the gas production rate with time (flux-time curves) at the macroscale. The simulation results show that the flux-time curves can be simplified to two linear segments in logarithmic coordinates, which are influenced by the fracture length and initial gas pressure. The predicted results using the PNM-FEM were validated by comparing them with the reported field test data. The method described in this study can be used to upscale the gas transport process from micro- to macroscale, which can provide a predictive tool for the gas production in shales.

## Introduction

Shales are the most common sedimentary rocks in the crust of the earth. Recent activities in shale gas exploration have shown that shale gas will be the most significant portion of the future global energy supply as traditional reserves begin to decline^1–4^. Improved reservoir conditions, advancement in horizontal drilling, and hydraulic fracking technology make shale gas extraction commercially feasible and successful^5, 6^. However, in contrast to traditional reservoirs, shale gas reservoirs appear to be more costly to develop and require specific technology for gas to be cost-effectively extracted due to the extremely low matrix permeability and porosity of shale^7, 8^. Thus, it is essential to accurately model the shale gas output to determine how quickly the gas can be extracted to generate revenue from each well and predict the economic feasibility of natural gas extraction.

The nanoscale pore structure and low permeability of shale cause the gas flow in shale to be non-Darcy, and change in pore morphology can significantly vary the gas flow^9^. In recent decades, various model has been developed to simulate the gas flow in shale. Mehmani, et al.^10^ report a multiscale and Multiphysics pore network model and their results showed that the nano-pores and pore connectivity have a major impact on the gas permeability. Zhang, et al.^11^ develop a three-dimensional pore network model considering the viscous flow, slip flow and Knudsen diffusion in shale, and their results showed the impact of gas pressure in pores, pore sizes and pore throat sizes. Zhang, et al.^12^ improve the above model by incorporating the anisotropy of shale and showed its influence on gas permeability. However, most of these models are valid at microscale and the length of the simulation ranges only several micrometers, which limits the real-world application of these models. Please note that experimental tests and field extraction are usually at the macro scale and the lengths of such samples are several orders larger than that in the scale of REV. Therefore, the predictions of pore or nanoscale models must be converted to engineering scale when modeling this engineering problem.

One of these parameters is the permeability of the porous medium, which describes the resistance to fluid flux in the porous medium in a representative volume. The concept of permeability does not necessarily require a detailed description of the fluid–solid interface at the cost of losing details on the pore scale. The contribution to shale gas generation depends on the gas pressures in trapped voids and the gas flow depends on hydraulic properties determined by the pressure gradients, which is also essential for computational viability^13^. Hence the influence of the pore structure of shales and the gas pressures of trapped voids cannot be ignored.

The different control factors of formation and gas exploration, such as intact shale gas pressure and fracture spacing, determine the gas production rate. There is limited research on the variables that control the actual shale gas production and their contribution because of the variability of those in unconventional shale gas deposits. Mayerhofer, et al.^14^ used a numerical simulation of explicit fracture networks to predict reservoir volume. Mattar, et al.^15^ proposed a theory and methodology based on a power-law exponential model. Their results showed the effect of the changing shutdown pressure distribution in the fracturing system on the volume flow rate of the well and the gas recovery. However, there is limited analysis of the flow rate and decline in production rate with time coupled with the pore structure of the shales. Besides the pore structures, facture length and initial pressures, other shale reservoir heterogeneities like components in shale (kerogen), adsorption/desorption in shale and pore fluid composition (dry gas) in shale can also influence the prediction of gas production^4, 16–18^.

This study aims to predict shale gas production using a micro–macro analysis method. For the microscopic analysis, four types of shales were considered, those included Eagle Ford (USA), Haynesville (USA), Longmaxi (China) and Opalinus (Germany). The microscale permeability-pressure relations (*K*-*p*) for the above four shales were established using the equivalent anisotropic pore network models (PNMs) based on the tested pore structure parameters obtained from representative shale samples. Anisotropic shale structure characteristics and the multiple migration mechanism including viscous flow, slip flow and Knudsen diffusion were coupled in the PNM. The *K*-*p* correlations were then used in a finite element model (FEM) to upscale the gas production at the macroscale in fractured horizontal wells. This FEM was used to predict the shale gas output under various reservoir scenarios and investigate the factors that influence the declining trend of shale production rate over time. The findings discussed here would enable a better understanding of gas production and depletion in shale gas wells under actual reservoir conditions.

## Microscale: pore network model simulations

The representative units of the equivalent pore network models were constructed for four different shale formations to determine the *K*-*p* correlations for different shale matrices.

### Flow calculation in pores

When gas flows in nanoscale pores, the velocities of gas molecules at the sidewalls of the pores were not the same^19^. Therefore, Klinkenberg first studied the gas slip phenomenon in porous media and found that the slip effect significantly influenced gas flow in porous media and introduced an expression for the gas flow correction factor (*F*) considering the slip^20^:

1 $$F\,{ = }\,1\, + \,\frac{{b_{{\text{k}}} }}{p}\, = \,1\, + \,\frac{{4\overline{\lambda } }}{r},$$

where *b*_k_ is the Klinkenberg slip factor which is due to the slip flow of gas at pore walls which enhances gas flow when pore sizes are very small, *p* is the gas pressure, *r* is the characteristic dimensions of flow channels, $\overline{\lambda }$ is the mean free path of gas molecules.

In this study, the Klinkenberg slip factor was introduced to modify Darcy’s law to incorporate the gas slip effect, and the mass flow *Q*_m_ can be presented as:

2 $$Q_{m} \, = \, - \,F\,\frac{{\rho k_{0} }}{{\mu_{\text{g}} }}\,\nabla p\, = \, - \,F\,\frac{M}{RT}\,\frac{{pk_{0} }}{{\mu_{\text{g}} }}\,\nabla p,$$

where *ρ* is the density of the gas, *k*_0_ is the intrinsic permeability of the shale, *μ*_g_ is the gas viscosity coefficient, *M* is the gas molar mass, *R* is the ideal gas constant, *T* is the absolute temperature.

The diffusion of gas molecules in the nanotube in the PNM is mainly caused by the violent collision with the sidewalls of pore throats. The Knudsen diffusion is often introduced to describe this phenomenon. The mass flow rate *Q*_d_ of gas molecules produced by diffusion can be described using Fick’s law:

3 $$Q_{d} \, = \, - \,D_{i} \frac{\partial c}{{\partial x}},$$

where *D*_i_ is the diffusion coefficient of the gas in the channels, and *c* is the mass fraction of gas, *x* is the distance.

Gilron and Soffer^21^ proposed that the diffusion coefficient *D*_i_ was composed of the modified diffusion coefficient *D*_i,c_ and the thermodynamic correction factor *ψ*(*T*,*p*), namely:

4 $$D_{i} \, = \,D_{i,c} \,\psi \, = \,\frac{\partial \ln p}{{\partial c}}.$$

Therefore, substituting Eq. (4) into Eq. (3) can obtain the diffusion mass flow rate. In Eq. (4), replacing *D*_i,c_ with the Knudsen diffusion coefficient can obtain the Knudsen diffusion mass flow rate. The Knudsen diffusion coefficient is:

5 $$D_{k,c} \, = \,\frac{2r}{3}\,\sqrt {\frac{8RT}{{\pi M}}} .$$

The simultaneous combination of Eqs. (3), (4), (5) can be used to obtain the mass flow rate of gas flow in a single tube due to Knudsen diffusion:

6 $$Q_{\text{k}} \, = \, - \,\frac{2r}{3}\,\sqrt {\frac{8RT}{{\pi M}}\,} \frac{M}{RT}\,\nabla p.$$

Here the Klinkenberg slip flow and Knudsen diffusion terms were accounted for to modify the mass flow and Eq. (6) accounts for the transition between flow regimes determined by the change of the Knudsen number. Hence the revised mass flow *Q*_m_ can be calculated by Eq. (7):

7 $$Q_{m} \, = \, - \,\left( {F\,\frac{{pr^{2} }}{{8\mu_{{\text{g}}} }}f\, + \,\frac{2r}{3}\sqrt {\frac{8RT}{{\pi M}}} \,\left( {1 - f} \right)} \right)\,\frac{M}{RT}\,\nabla p,$$

where *F* is the gas flow correction factor, of which the definition has been shown in Eq. (1), *p* is the gas pressure, *r* is the characteristic dimensions of flow channels, *μ*_g_ is the gas viscosity coefficient, *M* is the gas molar mass, *R* is the ideal gas constant, *T* is the absolute temperature. The *f* in Eq. (7) is the ratio of collisions between gas molecules and the pore throats, which can be determined by the following equation^22^:

8 $$f\, = \,\frac{1}{{1 + 0.5K_{{\text{n}}} }}\, = \,\frac{1}{{1 + 0.5\frac{{\overline{\lambda } }}{r}}},$$

where the *K*_n_ is the Knudsen number.

### Gas transport between pores

The dynamic pore-scale flow with multiple flow mechanisms is converted into a function of a single variable pore pressure, which is convenient for the time and space discretization of the pressure term in the calculation. Therefore, the dynamic pore-scale flow calculation requires the pore-scale discretization of the continuum, including each pore throat that contributes to the micro-scale gas flow^11^.

The gas flow in the pore network model is a dynamic process, and each pore needs to satisfy the law of conservation of mass. Hence select pore *i* as a reference, and pore *i* is connected to the surrounding *n* pores. At each time step, the pore throat pressure is equal to the mean value of the pore pressures connected with it. The mass change of pore *i* from time step *k* to time step *k* + 1 can be written as^11^:

9 $$m_{i}^{k} \, - \,m_{i}^{k + 1} \, = \,\sum\limits_{n} {\frac{M}{RT}\,\left( {F\,\frac{{\pi r_{{{\text{t}} {\text{h}} }}^{4} }}{{8\mu_{g} }}\,\,\frac{{\left( {p_{i}^{k} + p_{j}^{k} } \right)}}{2}\, + \,\frac{{2r_{th} }}{3}\,\sqrt {\frac{8RT}{{\pi M}}} } \right)} \,\frac{{p_{i}^{k} - p_{j}^{k} }}{{l_{{{\text{th}}}} }}\,\Delta t,$$

where *r*_th_ and *l*_th_ are the radius and the length of the pore throat between pores *i* and *j*. For ideal gas, the mass of gas stored in pore *i* in time step *k* is:

10 $$m_{i}^{k} \, = \,\frac{4}{3}\,\pi r_{i}^{3} \,\frac{M}{RT}\,p_{i}^{k} .$$

Substituting formula (10) into formula (9):

11 $$p_{i}^{k + 1} \, = \,p_{i}^{k} \, - \,\sum\limits_{n} {\left( {F\frac{{\pi r_{th}^{4} }}{{8\mu_{\text{g}} }}\,\frac{{\left( {p_{i}^{k} + p_{j}^{k} } \right)}}{2} + \frac{{2r_{th} }}{3}\sqrt {\frac{8RT}{{\pi M}}} } \right)\,} \frac{{p_{i}^{k} - p_{j}^{k} }}{{l_{{{\text{th}}}} }}\,\Delta t/\frac{4}{3}\pi r_{i}^{3} .$$

Equation (11) shows the dynamic iterative process of the pore pressure term. In the dynamic calculation, the time step is crucial for the stability of the explicit solution. In this study, the time step can be determined by the Courant number dynamically, which can be expressed as^11^:

12 $$\frac{v\Delta t}{{\Delta l}}\,{ = }\,\frac{{\frac{{r_{{{\text{th}}}}^{2} }}{{8\mu_{g} }}\frac{{p_{{\text{i}}}^{{\text{k}}} - p_{{\text{j}}}^{{\text{k}}} }}{{l{\text{cb}}}}\Delta t}}{{l{\text{cb}}}}\, \le \,1,$$

where the *l*_cb_ value is the length of the coordination bond between two adjacent pores and Δ*t* is the time step for maintaining stable flow calculation between adjacent pores. Thus, the time step will be calculated for all pores in the entire pore network, and the minimum value of the time steps for all pores was selected as the time step for the entire pore network model. In other words, in this calculation, the Courant number was kept less than 1 to ensure the stability of the iteration. Therefore, it was necessary to calculate the maximum time step allowed for all pores and take the smallest value for the simulation. Hence the calculation steps are as follows:

- (1) According to the initial calculated conditions, assign initial pore pressures to all pores.
- (2) Adjust the pressure value for the pores at the boundary according to the calculated boundary conditions: for constant pressure boundary conditions, update the pore pressure to the set pressure value; for closed boundary conditions, keep the previous iteration result without processing.
- (3) Calculate the Courant number of all pores and obtain the maximum allowable iteration time step under the condition that the Courant number is less than 1.
- (4) According to the iteration time step, use Eq. (11) to calculate the pore pressure for the next iteration step for all pores.
- (5) Repeat steps (2)–(4) until the iteration is completed or the convergence is reached.

### Development of a pore network model for shale formations

**Figure 1:**
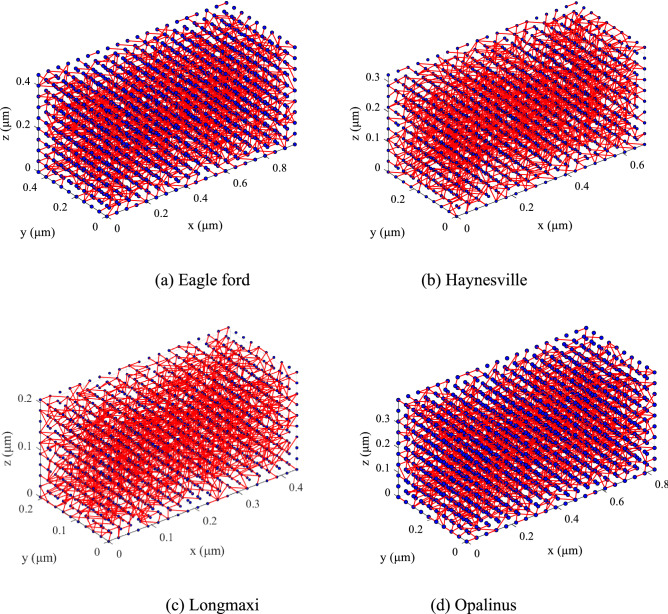
The schematic diagrams of the PNMs of the four types of shales.

Four anisotropic pore networks were developed for the four shale formations studied in this research using the steps mentioned above. The size of each anisotropic pore network was 20 × 20 × 20. The pore diameters and porosity values were obtained from the experimental data presented before and shown in Table 1. In a previous study, different tests (X-Ray Diffractometer Analysis (XRD), Scanning Electron Microscope (SEM) Analysis, Thermo Gravimetric Analysis (TGA), Computer Technology (CT) and Pore Size Distribution Analysis using the Brunauer–Emmett–Teller (BET) gas adsorption method) were conducted to obtain pore structures of the four shale formations studied in this research under laboratory conditions^23^. Other parameters, including the coordination numbers and anisotropic ratios, were set based on previous research on shale^12^. The schematic diagrams of the PNMs of the four types of shales were shown in Fig. 1, where the pore throats were simplified as lines.

**Table 1:** Shale sample parameters^23, 24^.

Sample	Pore radius /standard deviation (nm)	Pore throat radius/standard deviation (nm)	Coordination number/standard deviation	Anisotropic ratio (*a*_x_:*a*_y_;*a*_z_)	Porosity (%)
Eagle ford	8.5/0.4	2.9/0.2	4/0.1	22:22:1	4.41
Haynesville	5.4/0.3	1.6/0.1	4/0.1	22:22:1	2.94
Longmaxi	4.3/0.3	0.66/0.1	4/0.1	22:22:1	3.47
Opalinus	9.8/0.5	3.1/0.3	4/0.1	22:22:1	7.81

### Permeability-pressure correlations

**Figure 2:**
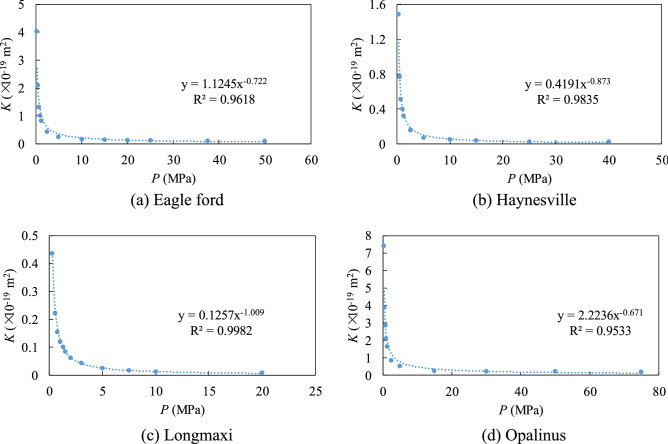
*K*-*p* correlations of the four types of shales.

Using those four anisotropic pore networks developed, the correlations between gas permeability and gas pressure were established. Linear initial pressure distributions were set along the model, and constant pressure boundaries were set at the left and right boundaries. The permeability of the gas is then calculated based on the following equation:

13 $$k\, = \,\frac{{2\mu_{{\text{g}}} RT}}{{\left( {p_{{\text{l}}} + p_{{\text{r}}} } \right)M}}\,\frac{{L_{{{\text{PNM}}}} }}{{A_{{{\text{PNM}}}} }}\,\frac{{Q_{m} }}{{\left( {p_{{\text{l}}} - p_{{\text{r}}} } \right)}},$$

where *p*_l_ and *p*_r_ are the constant boundary pressure at the left and right boundary of the PNM, *L*_PNM_ and *A*_PNM_ are the length and section area of the PNM respectively. Then the correlations between the average pressure in the model and the permeability are obtained and the results are shown in Fig. 2.

Figure 2 shows that the *K-p* correlations of the four shales can be fitted by power functions with *R*^2^ > 0.95. When *P* < 10 MPa, the permeability of the gas dropped rapidly with pressure and when *P* > 10 MPa the permeability was almost stable. Above results implied that the permeability of the shale is significantly influenced by the pore structure. Shale matrix with larger pore and pore throat sizes has higher permeability values when the pressure was the same.

## Macroscale: finite element model simulations

The finite element model (FEM) was then used to upscale the gas transport in shales from micro- to macroscale. The *K*-*p* correlations were coupled in the model to account the multiple transport mechanisms which had been considered in the PNMs. The change in gas production rate with the time for the shale fractures was obtained using the FEM and the influence of initial pressure and fracture length on the change in gas production rate with the time was then discussed.

### FEM settings

A one-dimensional FEM was used to simulate the gas production in fractures. The governing equation of gas mass balance in half of the fracture is shown below:

14 $$\frac{\partial }{\partial t}\,\left( {\theta \rho } \right)\, + \,\frac{\partial }{\partial x}\,\left( {\rho u} \right)\, = \,0,$$

15 $$u\, = \, - \,\frac{K}{{\mu_{{\text{g}}} }}\frac{\partial p}{{\partial x}},$$

where *θ* is the porosity of the shale, *ρ* is the density of the gas, *u* is the velocity of the gas, *K* is the permeability of the gas, which was coupled to pressure based on the correlations shown in Fig. 2.

**Figure 3:**
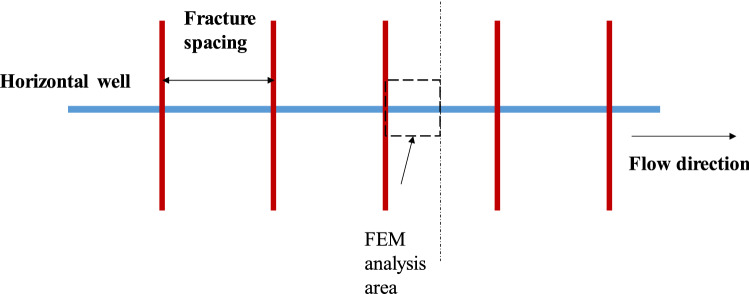
Schematic diagram of the horizontal well and fractures.

To simulate the gas production in the fracture, the schematic diagram shown in Fig. 3 was used, and half of the fracture was considered for analysis. A total of 500 elements were used for the FEM simulation. Constant initial pressure was assigned to all elements, which was 37 MPa for Longmaxi shale as shown in Table 2. A no-flux or reflection boundary was set at the left boundary and a constant pressure equal to wellbore pressure was set as the right boundary (outlet). The parameters used in the FEMs are shown in Table 2 according to previous field studies. The governing equations were then solved by a FEM module in the COMSOL Multiphysics 5.6 software using backward differentiation. The implicit time step was determined by using a local error estimate with a tolerance value of 0.1.

**Table 2:** Input parameters for selected four major shale formations^2, 12, 24–28^.

Input parameter	Eagle ford*	Haynesville*	Longmaxi	Opalinus*
Initial reservoir pressure, pin (MPa)	49	38	37	54
Wellbore pressure, pout (MPa)	0.1	0.1	0.1	0.1
Fracture length (m)	40	23	27	40
Reservoir temperature (^o^K)	323.4	350	356	356

*The reported values were best estimated based on literature.

### The impact of initial pressure and fracture length

**Figure 4:**
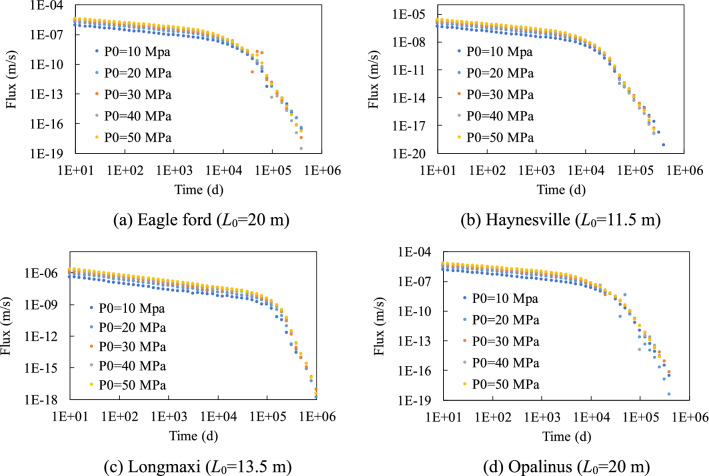
Influence of initial pressure on the gas production rate.

**Figure 5:**
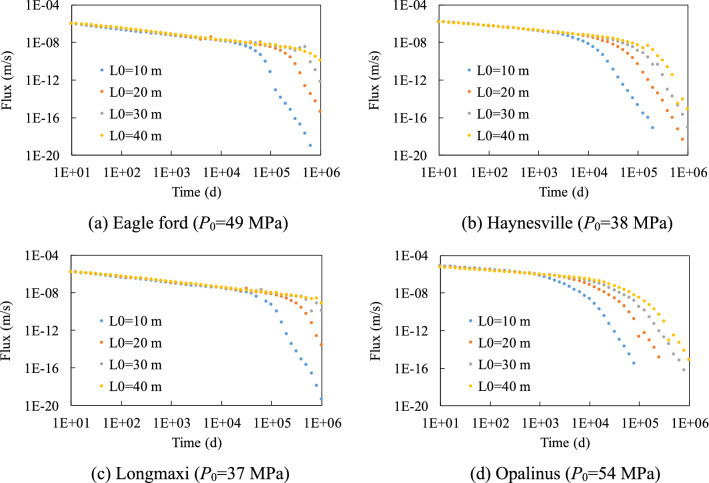
Influence of half fracture length on the gas production rate.

**Figure 6:**
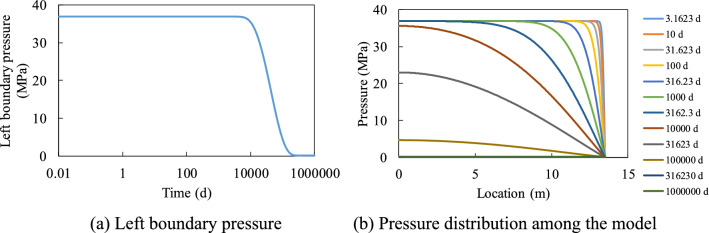
Change of pressure with time in the model (Longmaxi shale, *L*_0_ = 13.5 m, *P*_0_ = 37 MPa).

Figures 4 and 5 show the gas production rate with time under different initial pressure *P*_0_ values and half fracture length *L*_0_ values. Results imply that the flux-time curves show two-linear segments in logarithmic coordinates, and this can be explained by the change of pressure distribution in the model with time, of which one example is shown in Fig. 6. During the production, the pressure near the right boundary started to drop and this pressure drop passes to the left boundary gradually. Before the pressure drop reached the left boundary, the pressure of the left boundary pressure maintains the initial pressure, which corresponds to the first linear segment in logarithmic coordinates. After the pressure drop reached the left boundary, the pressure of the left boundary starts to decrease and this leads to a faster decrease of pressure for the whole model, which corresponds to the second linear segment in logarithmic coordinates.

Both initial pressure and fracture length can influence gas production. Higher initial pressure leads to a higher gas production rate in the first segment. The flux-time curves can be normalized by initial pressure in the first segment since the pressure distribution in the model is proportional to the initial pressure. The influence of pressure in the second segment is complicated and cannot be normalized by initial pressure, which shows the nonlinear contribution of pressure on permeability (Fig. 2).

Figure 6 shows the influence of half fracture length on the gas production rate. The flux-time curves of the model with different fracture lengths overlapped during the first stage because the pressure drop has not reached the left boundary. A longer model shows a longer first stage and can assume that if the model is infinite in length, then the flux-time curve will only have the first linear stage. The slope of the second stage increases with the model length but was always smaller than the slope of the first stage in logarithmic coordinates.

### Comparison with field data

**Figure 7:**
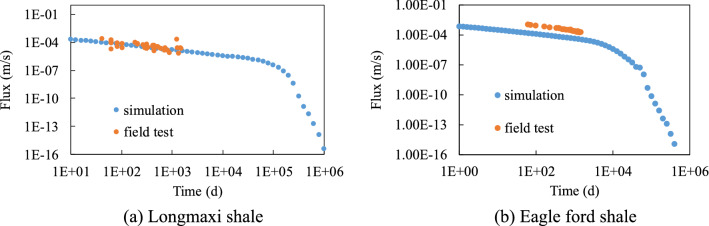
Comparison between simulation results and field test data (Longmaxi shale and Eagle ford shale).

The comparisons between simulation results and actual field data from Longmaxi shale and Eagle Ford shale are shown in Fig. 7. This was based on field test data reported by Wei, et al.^29^ and Baihly et al.^2^. The gas-bearing layer in the lower part of the Longmaxi shale formation has a thickness of 30 m. The simulation results of the half structure need to be multiplied by the number of half fractures in the horizontal well. Based on the field performance data for the Longmaxi shale, the length of the horizontal well was 1800 m for the reported field test data. According to Cusack, et al.^30^, the thickness of Eagle ford shale can range from 125 to 320 ft. Here, the average value of the thickness is set as 222.5 ft (67.8 m). For Eagle ford shale, the engineering parameters like the thickness of the shale and the length of the horizontal well have not been included in their publication. The length of the horizontal well is difficult to obtain from reported data, which is usually based on engineering experience. Hence the same length as that for Longmaxi shale (1800 m) was used in the simulation, which may be the reason for the slight deviation.

Figure 7 shows the change of flux with time obtained from simulation is similar to the reported field test data. The simulation results of Longmaxi shale are in good agreement with the field test data. The minor discrepancy between the simulation results of Eagle Ford shale and the field test data may be due to the uncertainty of the engineering parameters. Results show that the prediction of the simulation results using the micro–macro method provided by this study can be an effective method to predict the flow rate decline with the time of shale formation. The simulation results were not included for the other two shale formations as the required FEM parameters were not available in the literature.

## Discussion

The results provided by this study can be used to predict the transport of shale gas under different scales under in-situ conditions or laboratory conditions.

At the pore scale, the permeability of the shale gas decreases with pressure (Fig. 2). Similar behavior has been reported based on experimental measurements^31^. Zhang et al.^12^ show that the PNM can effectively predict the laboratory test results if the pore structure parameters (pore radius, pore throat radius, coordination number, porosity and anisotropic ratio) are known from nano/micro-scale tests. In this study, the PNM was first used to predict the *K*-*p* correlations for four shales. The predicted *K*-*p* correlations depend on the pore structure of shale and the multiple transport mechanisms of shale gas. To use this correlation in the upscale model, a power function was fitted to the predicted *K*-*p* correlations and used in the FEM simulations.

At the fracture scale, the variation of gas production rate with time (flux-time curves) can be obtained by the FEM model (Figs. 4 and 5). Please note that the PNM was usually used to study microscopic mechanisms at micro scales and was computationally very expensive at the macroscale. Hence in this study FEM model was coupled to the *K*-*p* correlations to solve this problem. The results of this study showed that the flux-time curves can be divided into two stages in logarithmic coordinates, which are driven by the change of pressure distribution among the fracture. Previous studies have reported similar power law of an exponential model to describe the flux-time curves^15^. Figures 4 and 5 also show that when the simulation runs for a long time during the gas production, the decrease of flux with time will change as soon as the gas pressure at the far end boundary change. The inflection point was reached at that time and then the gas production rate rapidly declined. In addition, the gas production rate depends on the initial gas pressure and fracture length. Under higher initial pressure, the initial gas flux rate was higher, and there was a delay in the inflection points for long fracture lengths.

For horizontal wells, the method described in this study can be used to predict gas production if the length of the well and number of the fractures are known. Figure 7 showed that the gas production of Longmaxi shale and Eagle Ford shale can be predicted by this method.

**Figure 8:**
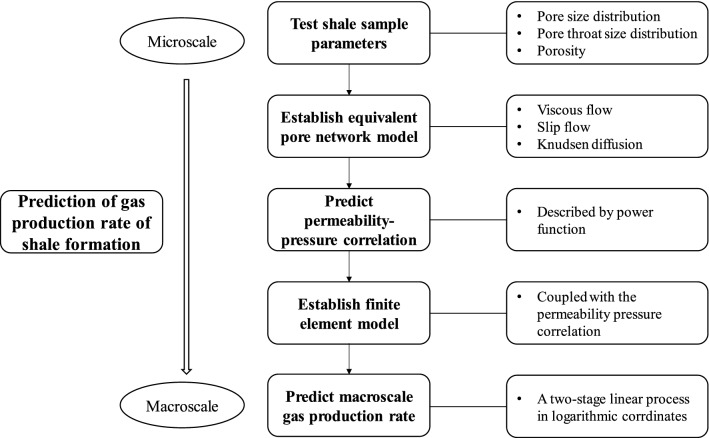
The flowchart of the micro–macro methodology to predict the gas production rate of shale formation.

A flowchart of the proposed methodology is shown in Fig. 8, which can be used the other shale deposits once accurate field data for those formations are available. The method can be implemented to predict actual gas production in horizontal wells using the flow chart shown in Fig. 8 and can be used for further studies of unconventional shale gas reservoirs.

The methodology described in this manuscript to predict the gas production rate is a simple one based on micro/nano pore structure and does not consider complexities of gas-bearing shale such as pore components (kerogen), processes (absorption and desorption) and pore fluid composition (remaining fracture fluids or gas condensate). However, this is a first attempt to predict the actual gas production based on micro-nano pore structure of shale. The research team currently is working further expand the methodology described in this study to include other contributing complexities of gas-bearing shale such as pore components, processes and pore fluid composition^4, 16–18, 32–34^.

## Summary and conclusions

The prediction of gas production rates after fractures and their declining gas production patterns for unknown shale reservoirs is important for a full recovery and economic decision-making. This research first introduced a micro–macro pore network method considering the pore structure of the shale sample to estimate the gas production and the decline trends for shale formations. The pore structure was described by an anisotropic equivalent pore network model coupling the multiple gas transport mechanism, which can provide the variation of the permeability-pressure correlations for a given shale formation. The variation of the permeability-pressure correlations was then applied to a finite-element model to upscale the gas production from micro- to macroscale. The influence of initial gas pressure and fracture length on the gas production process was analyzed. The effectiveness of the method was validated by field test data of Longmaxi shale and Eagle Ford shale. The major conclusions of this study were as follows:

- 1. The anisotropic pore network model with nanoscale gas diffusion is appropriate to represent shale gas reservoir production. In addition, the decline curve of permeability with gas pressure can be described by a power function.
- 2. Fracture length and initial gas pressure are shown to be the dominating factors that control the decline in gas flow. The flux-time curves have two-linear segments in the logarithmic coordinates, and this can be explained by the change of pressure distribution in the model with time.
- 3. A methodology was established to predict the gas production rates with time for unknown formations with limited field data. Hence this study lays the groundwork for scaling up the nano-scale pore network model for the estimation of engineering-scale shale gas production.

## Data Availability

Data will be made available on request by contacting the corresponding authors Liming Hu and Jay Meegoda.
